# Endosymbiont genomes yield clues of tubeworm success

**DOI:** 10.1038/s41396-018-0220-z

**Published:** 2018-07-18

**Authors:** Yuanning Li, Mark R. Liles, Kenneth M. Halanych

**Affiliations:** 1 Department of Biological Sciences & Molette Biology Laboratory for Environmental and Climate Change Studies, Auburn University, Auburn, AL 36830 USA; 2 Department of Biological Sciences, Auburn University, CASIC Building, Auburn, AL 36849 USA

**Keywords:** Molecular evolution, Comparative genomics

## Abstract

Forty years after discovery of chemosynthetic symbiosis in the tubeworm *Riftia pachyptila*, how organisms maintain their unique host–symbiont associations at the cellular level is still largely unknown. Previous studies primarily focus on symbionts associated with host lineages living in hydrothermal vents. To understand physiological adaptations and evolution in these holobiont systems in markedly different habitats, we characterized four novel siboglinid-symbiont genomes spanning deep-sea seep and sedimented environments. Our comparative analyses suggest that all sampled siboglinid chemoautotrophic symbionts, except for frenulate symbionts, can use both rTCA and Calvin cycle for carbon fixation. We hypothesize that over evolutionary time siboglinids have been able to utilize different bacterial lineages allowing greater metabolic flexibility of carbon fixation (e.g., rTCA) enabling tubeworms to thrive in more reducing habitats, such as vents and seeps. Moreover, we show that sulfur metabolism and molecular mechanisms related to initial infection are remarkably conserved across chemoautotrophic symbionts in different habitats. Unexpectedly, we find that the ability to use hydrogen, as an additional energy source, is potentially more widespread than previously recognized. Our comparative genomic results help elucidate potential mechanisms used to allow chemosynthetically dependent holobionts adapt to, and evolve in, different environments.

## Introduction

**Fig. 1:**
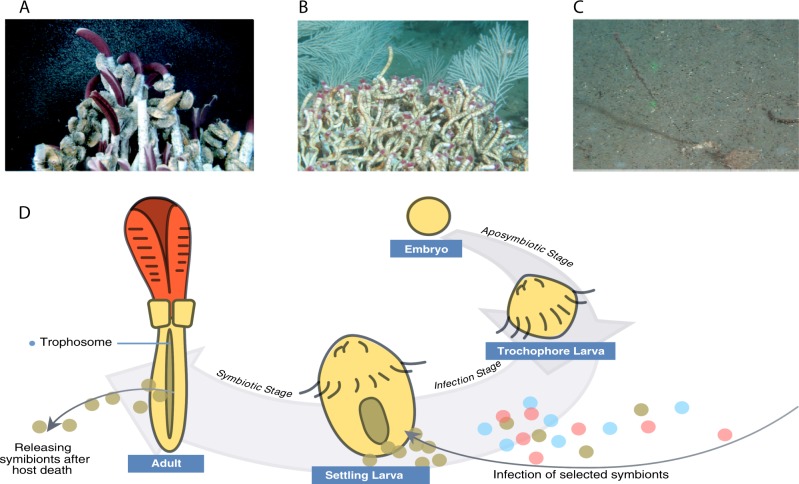
Major siboglinid lineages and life cycles associated with horizontally transmitted symbionts. **a** Giant tubeworm *Riftia* growing in hydrothermal vent (Image courtesy of Tim Shank from Woods Hole Oceanographic Institution). **b** *Lamellibrachia* growing near a hydrocarbon seep. **c** *Galathealinum* growing in deep-sea muddy habitats. **d** The different life stages of siboglinids associated with horizontally transmitted symbionts (modified from Fig. 2c of Bright and Bulgheresi [12] with updated understanding of siboglinid-symbiont transmission mode). The embryo and larval stage are aposymbiotic. Symbionts infect the settled larval skin, and then migrate to mesoderm that later will develop into trophosome. Symbionts are released upon host death. Environmental bacteria are shown in blue and pink

Since the discovery of the gutless *Rifita pachyptila* at hydrothermal vents near the Galapogos in 1977, scientists have realized that chemosynthetic symbioses between marine invertebrates and bacteria are ubiquitous in natural ecosystems, ranging from hydrothermal vents, cold seeps, organic falls, mud volcanoes to shallow water sediments [1]. Chemosynthetic symbioses can facilitate specialized deep-sea communities and have important roles in maintaining alpha and beta biodiversity, thereby facilitating adaptive radiation and evolutionary novelty. To date, at least seven invertebrate phyla have been characterized with chemosynthetic symbioses [1, 2]. Among them, siboglinid tubeworms, especially vestimentiferans that can attain sizes up to 2 m long and 5 cm in diameter, have drawn considerable attention because of their dominance and high levels of productivity at hydrothermal vents and cold seeps (Fig. 1a, b) [3]. As currently recognized, more than 200 species of siboglinids have been described within four lineages (Vestimentifera, Monilifera, *Osedax*, and Frenulata) [4, 5]. In comparison with vestimentiferans, frenulates are typically more diminutive (10 cm long but > 0.2 cm in diameter) and are often found in deep-sea muddy sediments (Fig. 1c). Previous phylogenetic analysis has shown different lineages of siboglinids host specific lineages of symbionts using 16S ribosomal RNA sequences [6]. Current hypotheses suggest that uptake and retention of sulfur-oxidizing symbionts from surrounding habitats allowed tubeworms to exploit and adapt to new habitats and resources; hosts may be able to selectively uptake bacteria with the most appropriate physiology for host in different habitats [7, 8]. However, genomic bases for physiological mechanisms allowing successful colonization of different environments have not been studied.

Most siboglinids are generally associated with a single ribotype of sulphur-oxidizing γ-Proteobacteria (but dual symbiont ribotypes have been found in *Lamellibrachia anaximandri* [9]) that reduce sulfur compounds as electron donors and fix CO_2_ autotrophically. Additional symbiont metabolic diversity has been discovered among siboglinids, such as the methanotrophic symbionts found in *Siboglinum poseidoni* [10], or the heterotrophic Oceanospirillales harbored by *Osedax* [11]. Siboglinid endosymbionts are obtained each generation through horizontal transmission of free-living symbionts from the surrounding environment presumably after larval settlement [8] (Fig. 1d). Symbionts are acquired horizontally during a symbiont-specific infection process after which they migrate into mesodermal tissue that will later develop into the trophosome, a specialized symbiont-containing organ [8, 12]. Upon death of the host, symbionts are released back into the environment enriching free-living populations [13]. Although symbiont transmission between generations is crucial for the persistence of the siboglinid-symbiont association, mechanisms underpinning this specialized infection process have not been fully characterized.

To date, only *Osedax* [11] and several vestimentiferan symbiont genomes (e.g., refs. [14–16]) have been sequenced and characterized. These recent genomic and proteomic studies suggested that vent-dwelling vestimentiferan symbionts are able to use the reductive tricarboxylic acid cycle (rTCA) in addition to previous identified Calvin–Benson–Bassham (CBB) cycle for CO_2_ fixation [14, 17, 18]. Moreover, key enzymatic genes, RubisCO and ATP citrate lyase (ACL) type II associated with these carbon fixation cycles, were identified in *Lamellibrachi*a and *Escarpia* symbionts [16, 19]. Given these limited data, how metabolic machineries differ between endosymbionts of vent-dwelling and seep-living vestimentiferans, or between symbionts of vestimentiferans and their diminutive cousins, the frenulates, is not well understood. This lack of information is particularly surprising given that vestimentiferan tubeworms are dominant fauna in some seep environments (e.g., Gulf of Mexico) and frenulates are the most diverse and widely distributed lineage of siboglinids throughout the deep-sea. The lack of genomic information for symbionts associated with other siboglinid lineages hinders understanding of genetic mechanisms involved in adaptation of host–symbiont systems to different chemosynthetic environments.

This study was initiated to determine encoded functional differences that may contribute to the ecological success of seep and vent vestimentiferans relative to their mud-dwelling frenulate cousins. We therefore sequenced genomes from symbionts of three seep-dwelling vestimentiferans and one mud-dwelling frenulate and compared them to endosymbiont genomes from hydrothermal vent siboglinids. Our comparative analysis revealed differences in energetic pathways (e.g., rTCA) of siboglinid holobionts that may account for adaptation to different deep-sea chemosynthetic communities. The findings provide hitherto unknown evidence for the dominance of some chemosynthetic symbioses in specific environments, as well as insights as to which cellular mechanisms may be conserved across different environmental settings.

## Materials and methods

### Sampling collection, DNA extraction, and sequencing

Siboglinid specimens were collected from seep localities in the Mississippi Canyon at 754 m depth in Gulf of Mexico (N 28°11.58′, W 89°47.94′), using the* R/V Seward Johnson* and *Johnson Sea Link* in October 2009. All samples were frozen at 80 °C following collection. Trophosome tissue was dissected from each worm, and total genomic DNA was extracted using the DNeasy Blood & Tissue Kit (Qiagen) according to the manufacturer’s protocols. Sequencing of genomic DNA was performed by The Genomic Services Lab at the Hudson Alpha Institute in Huntsville, Alabama using Illumina (San Diego, California) 2 × 100 paired-end TruSeq protocols on an Illumina HiSeq 2000 platform.

### Genome assembly, completeness assessment

Illumina fastq reads were trimmed using Sickle (https://github.com/najoshi/sickle) (with the parameters: pe sanger –q 30 –l 100). Resulting metagenomic data were assembled de novo with several different assemblers: Ray Meta 2.2.0 [20] with *k*-mer = 31 (value chosen based on comparing a range of *k*-mer values relative to final assembly), IDBA-UD with “pre_correction” [21] and MetaPlatanus 1.03 with default settings [22]. To identify putative symbiont contigs, BLAST [23] was performed on contigs produced by each assembler using the *Riftia* symbiont genome (GenBank Accession: NZ_AFOC00000000.1 [14]) as the bait sequence. The identified bacterial clusters were filtered using a BLASTx search against host transcriptomic assembly [5] to remove false positives originating in the host genome. Followed the study of Gardebrecht et al. [14], short genomic assemblies (<500 bp) which resulted from repetitive genomic regions and potential contamination from hosts were excluded from the assembly. Symbiont assemblies from different assemblers were evaluated using Quast 4.5 [24] based on size N50, number of contigs, GC content, and level of completeness.

To ensure the relative symbiont purity in the assembly, 16S rRNA genes were extracted using BLASTn and subsequently blasted against the 16S rRNA gene database (http://greengenes.lbl.gov). To test whether there was evidence of multiple symbiont ribotypes within single hosts, raw paired-end reads were remapped to each of their respective 16S rRNA gene contigs using Bowtie2 using the “--very-sensitive” parameter [25], and then visualized in Tablet [26]. γ-Proteobacteria taxa used in the phylogenetic analysis were based on previous results [6] and downloaded from TREEBASE (http://www.Treebase.org). Sequences of each OG were then aligned using MAFFT [27] with the ‘-auto’ and ‘-localpair’ parameters and 1000 maximum iterations. Maximum likelihood analyses were performed in RAxML 8.2.7 [28] under GTRGAMAMA model with rapid bootstrapping of 1000 replicates (Supplementary Figure S1).

Completeness of obtained bacteria genomes was assessed using CheckM [29]. Completeness of genomes was estimated via presence of 106 essential single-copy genes proposed by Dupont et al. [30] using HMMER3 [31], requiring 70% length match for each Hidden Markov models (HMMs) of these genes, with the trusted cutoff as the minimum score (Supplementary Figure S2). An additional estimate of completeness for siboglinid symbionts was compared to 577 lineage-specific single-copy marker sets [29] from 112 γ-Proteobacteria genomes (Supplementary Table S1). Moreover, the average nucleotide identity (ANI) and average Amino Acid identity (AAI) values of all siboglinid-symbiont genomes was calculated using shell scripts of *ani-matrix.sh* and *aai-matrix.sh* implemented in the enveomics package [32], respectively (Supplementary Table S2).

### Genome annotation and pathway analysis

Extracted endosymbiont genomes were uploaded to the RAST server (http://rast.nmpdr.org/) [33] for annotation. Metabolic pathway analysis of newly sequenced and publicly available genomes was performed by using the KEGG2 KAAS genome annotation web server [34] and then visualized by the KEGG Mapper Reconstruct Pathway tool (http://www.genome.jp/kegg/tool/map_pathway.html). A BLASTp search of protein sequences from genome annotation against the Swiss-Prot database was used to search for proteins that were missing in the visualized KEGG pathway or RAST annotations.

Representative group 1 Ni, Fe hydrogenase large subunit sequences identified in previous study [35] were aligned with *Escarpia*, *Seepiophila*, *Lamellibrachia*, and *Galathealinum* symbiont hydrogenase sequences using MAFFT with the ‘-auto’ and ‘-localpair’ parameters and 1000 maximum iterations. The alignment was then trimmed using the default settings in Gblocks 0.91b [36] to remove ambiguously aligned regions. For phylogenetic analyses, ProtTest 3 [37] was performed to evaluate all evolutionary models under a BIC criterion. Maximum likelihood analyses were performed in RAxML under PROTGAMAMALG model with rapid bootstrapping of 1000 replicates (Supplementary Figure S3). GenBank accession for each sequence was next to the tips of each operational taxonomic unit (OTU) in the tree.

### Comparative genomic and phylogenetic analysis of siboglinid symbionts

**Fig. 2:**
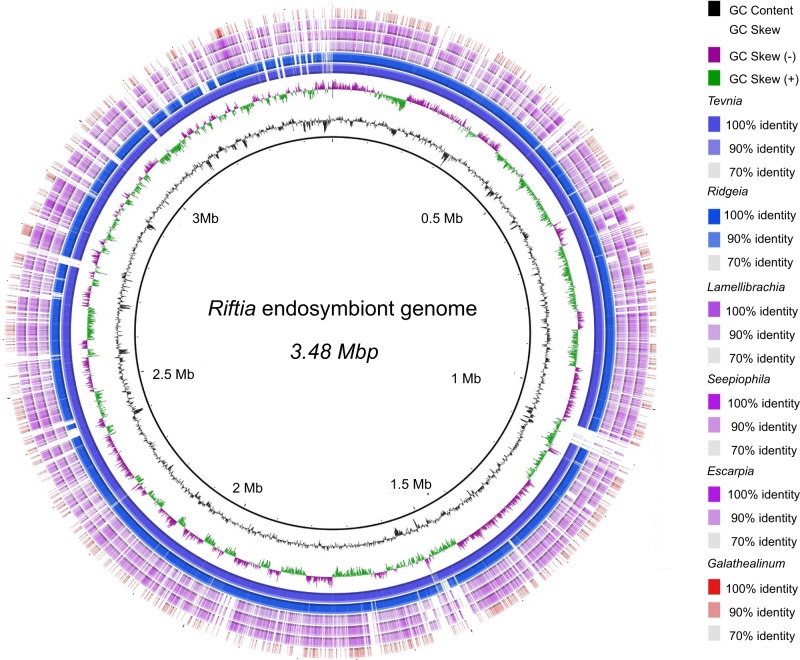
Whole-genome comparisons of sulfur-oxidizing symbionts from siboglinids. The inner circle represents the reference sequence, *Endoriftia* symbiont. The second and third circles show GC skew and %G+C, respectively. Inner rings correspond to assembled draft symbiont genomes found in vent-dwelling (blue) *Tevnia*, *Ridgeia*, seep-living (purple) *Lamellibrachia*, *Seepiophila*, *Escarpia,* and mud-dwelling *Galathealinum* (red). Genome identifiers are listed in order from inner to outer ring on the right of the legend. Figure was produced using BRIG

Three seep-living, one mud-dwelling, and three previously sequenced vent-dwelling siboglinid endosymbiont genome sequences were subjected to BLASTn comparisons for homologous regions of and then visualized using BRIG ([38]; Fig. 2). [Note: The genome sequences for endosymbionts of *Escarpia* and *Lamellibranchia* vestimentiferans living in low-temperature diffuse vent flow were recently published [16] while the present work was under review and thus are not included here]. To roughly characterize the protein composition of each siboglinid-symbiont genome, annotated genes were assigned to a RAST subsystem category and then plotted for comparison (Supplementary Figure S4).

Analysis of co-phylogeny was conducted from six siboglinids and corresponding endosymbionts; *Tevnia* was not included here due to the lack of a host transcriptome. A phylogenomic analysis, using highly conserved orthologous genes, compared the four newly sequenced genomes with other publicly available siboglinid-symbiont genomes. One-to-one orthologous genes (OGs) were generated using ProteinOrtho [39] with default parameters. Resulting OG clusters were then filtered from each bacterial genome following modified bioinformatics pipelines of [5, 40]. Briefly, all sequences shorter than 100 amino acid residues were discarded. Sequences of each OG were then aligned using MAFFT [27] with the ‘-auto’ and ‘-localpair’ parameters and 1000 maximum iterations. Uninformative and ambiguously aligned positions were trimmed with Aliscore [41] and Alicut [42]. Alignment columns with only gaps were subsequently removed, and any OG with an alignment less than 100 amino acid residues in length after trimming was discarded. For each OG, a custom java program, *AlignmentCompare.java* (https://github.com/kmkocot/basal_metazoan_phylogenomics_scripts_01-2015), was used to remove any sequence that did not overlap other sequences by at least 20 amino acids. For the host tree, the supermatrix dataset from selected taxa was derived from [5], yielding 289 OGs. For both datasets, OGs were concatenated into a single alignment using FASconCAT [43]. Maximum likelihood analyses were performed in RAxML under the best-fitting models for associated partition schemes determined by PartitionFinder with rapid bootstrapping of 100 replicates (Supplementary Figure S5B).

### GenBank accession and data repository

GenBank accession numbers for the siboglinid-symbiont genomes are provided in Table 1 (accession no. PRJNA454446). Phylogenetic datasets were deposited to figshare, figshare.com (10.6084/m9.figshare.6332288.v1).

**Table 1:** Overview of the siboglinid symbiot assembies

	Vent-dwelling vestimentiferans			Seep-living vestimentifernas		Frenulata	
Features^a^	*Riftia*	*Ridgeia*	*Tevnia*	*Escarpia*	*Lamellibrachia*	*Seepiophila*	*Galathealinum*
Genome size (Mb)	3.48	3.44	3.64	4.06	3.53	3.53	3.77
N50 (Kb)	29.6	83.9	92.7	313.6	20.6	20.6	726.7
Coverage (folds)	25	180	15	121	20	18	75.6
% G+C	58.8	58.9	58.2	54.2	54.3	54.3	38.9
No. of contigs	197	97	184	23	337	323	14
No. of predicted genes	3341	3188	3566	3698	3552	3542	3497
No. of RNA	45	50	47	48	48	48	43
Assembler	—	—	—	MetaPlatanus	Ray	Ray	MetaPlatanus
Accession	AFOC00000000	LMX100000000	AFZB00000000	QFXE00000000	QFXD00000000	QFXF00000000	QFXC00000000

^a^Data for symbionts of *Esarpia*, *Lamellibrachia*, *Seepiophila* and *Galathealinum* are from this study; data for *Riftia* and *Tevnia* are from the paper of Gardebrecht et al. [14]; data for *Ridgeia* is derived from the study of Perez and Juniper [15]

## Results and discussion

### Genomic features

Results from high-throughput sequencing and genome assembly for siboglinid symbionts are presented in Table 1. Estimation of genome completeness based on 106 essential bacterial single-copy genes and the resulting 577 lineage-specific core genes resulted in >95% overall completeness of newly sequenced symbiont genomes with a low level of contamination compared to other sequenced siboglinid-symbiont genomes (Supplementary Figure S2 and Table S1, respectively), indicating completeness of the predicted gene models. The *Escarpia* and *Galathealinum* symbiont assembly (23 and 14 contigs, respectively) contained significantly fewer and longer contigs in comparison with other sequenced siboglinid symbionts (Table 1) (Supplementary note S1). The comparison of AAI and ANI values are provided in Supplementary Table S2.

The BLAST comparison of these genomic contigs revealed relatively strong homology across siboglinid-symbiont genomes in line with their degree of relatedness (e.g., seep-dwelling vestimentiferan symbionts show greatest homology and the *Galathealinum* symbiont showed the least) (Fig. 2). In contrast, clusters of functional groups based on RAST subsystem predictions showed largely functional homogeneity among all sequenced siboglinid symbionts (Supplementary Figure S4).

Consistent with results from previous analyses that indicate most siboglinid species host a single symbiont ribotype [44], but see ref. [9], only one good hit (*e*-value cutoff: 1e–5) of a 16S rRNA gene sequence was recovered from each symbiont assembly using BLASTn against the Greengenes database [45], and no nucleotide sequence differences were observed via subsequent remapping of raw reads. Both results strongly support the conclusion that only one bacterial endosymbiont ribotype was present in any of the hosts examined in this study (see Supplementary note S2).

Cophylogenetic analyses (see Supplementary note S3) revealed an incongruent phylogeny between the hosts and symbionts (Supplementary Figure S5), consistent with previous research [6].

### Nutrients and metabolism

#### Carbon cycle

**Fig. 3:**
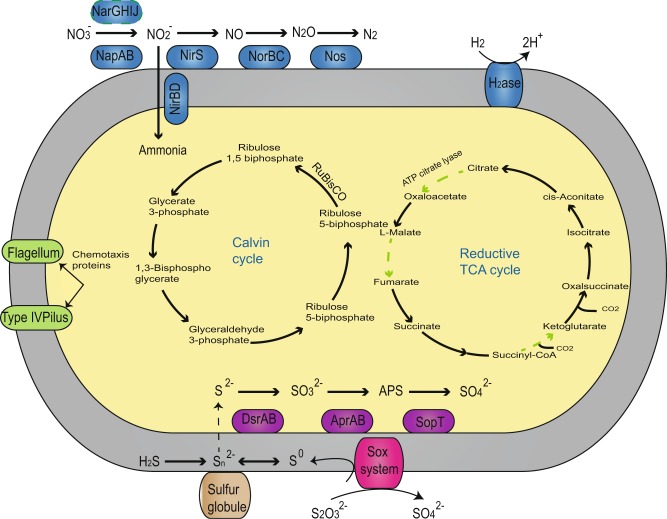
Overview of the major cellular features and central metabolism in the deep-sea sulfur-oxidizing siboglinid symbionts. Pathways for which no predictable enzymes were found in frenulate species *Galatehalinum* and seep-dwelling *Escarpia* symbiont genome are shown in green and red dash lines, respectively. Numbers of transport machineries are shown for both strains. The KEGG database was used for the reconstruction of metabolic pathways. *H2ase* hydrogenase, *nap* periplasmic nitrate reductase, *nir* cytochrome nitrite reductase, *nor* nitric oxide reductase, *nos* nitrous oxide reductase, *dsrAB* dissimilatory sulfite reductase, *AprAB* APS reductase, *APS* adenosine-5-phosphosulfate reductase, *sopT* ATP sulfurylase

All sequenced siboglinid-symbiont genomes contained core components of the Calvin–Benson–Bassham (CBB) cycle for carbon fixation (Fig. 3). Similar to previous sequenced *Riftia* symbiont species [14, 17, 18], all components of reductive tricarboxylic acid cycle (rTCA) were also found in *Lamellibrachia*, *Escarpia* and *Seepiophila* symbiont genomes, with enzymes in the TCA/rTCA pathways shared across the sampled taxa. However, three key enzymes that allow the cycle to run in reverse, ATP citrate lyase (*aclAB*), 2-oxoglutarate:ferredoxin oxidoreductase *(korB*), and Fumarate reductase (*sdhAC*) [46], were missing in the *Galathealinum* symbiont genome (Fig. 3).

The rTCA cycle demands significantly less energy than the CBB cycle and is generally considered the most energy-efficient CO_2_ fixation pathway [47]. However, several enzymes associated with rTCA cycle are highly oxygen sensitive compared to the CBB cycle; therefore, this limits the rTCA cycle to anaerobic and microaerobic bacteria that occur in certain anoxic or hypoxic environments (e.g., vents) [48]. Possession of the rTCA cycle in vestimentiferan symbionts may be important for symbionts’ heterotrophic stage since they must travel across the redox boundary to access both reducing compounds from anoxic habitats as energy sources and the oxygen from the water column for aerobic metabolism [49]. A previous study [14] reported that the spot volume in enzymes associated with rTCA was higher in *Tevnia* than *Riftia* symbionts owing to higher oxygen levels in the vent fluid surrounding *Riftia* than *Tevnia*. Symbionts with depleted energy sources (e.g. low sulfur content) may switch to using the rTCA cycle, which would allow for metabolic flexibility and thus facilitate adaptation in different environmental conditions [17, 50]. As suggested by Thiel et al. [19], the presence of rTCA in addition to CBB pathways for carbon might be common in all vent-dwelling and seep-living vestimentiferan endosymbionts. Presently, available data suggest that the CBB cycle is the predominant carbon fixation pathway in both seeps and vents, whereas thiotrophs using rTCA cycle are thought to be found only in symbionts from vent environments [50]. However, endosymbionts of seep-living vestimentiferans and some other polychaetes (e.g., the scale worm *Branchinotogluma sandersi*) also rely on the rTCA cycle for carbon fixation, thereby providing additional support for the widespread occurrence and importance of the rTCA cycle in seeps [51].

Although the *Galathealinum* symbiont genome contains all components required for the CBB cycle similar to vestimentiferans, key enzymes associated with the rTCA cycle were lacking. Frenulates are sister to all other siboglinid lineages [4, 5, 52] and mainly found in anoxic or hypoxic reducing muddy sediments with a relatively lower sulfur level compared to vents and seeps [53]. Although *Galathealinum* was collected in the muddy sediment nearby the seep locality, it harbored endosymbionts belonging to a different bacterial clade (Supplementary Figure S1 [6]). In addition, multiple copies of shared enzymes that function in both pathways were identified in all sequenced vestimentiferan symbiont genomes since they have been proposed to employ separate sets of genes for the oxidative and reverse direction of the TCA cycle [14]. In contrast, only one copy of these genes was discovered in the *Galathealinum* symbiont (e.g., Pyruvate:ferredoxin oxidoreductas (*porAG*) has four to five copies in vestimentiferan symbionts and only one copy in the *Galathealinum* symbiont). The presence of the rTCA cycle and multiple copies of associated genes involved in oxidation may result in higher rates of CO_2_ fixation among vestimentiferans, resulting in greater body and larger population size compared to frenulates. Along these lines, we hypothesize that over evolutionary time siboglinids have been able to utilize a bacterial lineages with a higher metabolic flexibility of carbon fixation in environments that allow them to successfully exploit and thrive in more reducing habitats, such as vents and seeps.

#### Sulfide and nitrogen metabolism

Previous work [14] has shown that vent-dwelling vestimentiferan symbionts are physiological homogeneous, but physiological differences in siboglinid symbionts isolated from other chemosynthetic habitats has not been assessed. Our results show that sulfur metabolic pathways are largely conserved across all siboglinid symbionts, suggesting that seep-living vestimentiferan and frenulate symbionts also oxidize sulfide to sulfate via a reverse sulfate reduction pathway for sulfide oxidation (APS reductase pathway; Fig. 3, Supplementary note S3).

Nitrate is extremely abundant in deep-sea hydrothermal vents and cold seeps [54, 55]. All four siboglinid-symbiont genomes were found to encode the entire set of enzymes of dissimilatory nitrate reduction and dissimilatory nitrate reduction to ammonium as a nutrition source for biosynthesis and growth [56]. Previous analysis [57] suggested that the majority of nitrate is reduced to ammonia instead of nitrogen gas for nitrate respiration in *Riftia* symbionts, although *Ridgeia* symbionts were capable of both dissimilatory nitrate reduction and ammonia assimilation [58]. Genes required to convert nitrite to nitrous oxide were found in all symbiont genomes (nitrite reductase—*nirK* and nitrite oxidoreductase—*norCB*). Unexpectedly, two types of nitrate reductases (i.e. membrane-bound respiratory nitrate reductase: *narGHIJ*; periplasmic dissimilatory nitrate reductase: *napABC*) that can catalyze reduction of nitrate to nitrite and their associated electron carriers were found in *Riftia*, *Tevnia*, *Lamellibrachia*, and *Seepiophila* symbionts, whereas only the *nap* operon was found in *Escarpia* and *Galathealinum* symbiont genomes (Fig. 3). The *nap* system used in the *Riftia* symbiont has been proposed to compensate *narGHIJ* and perform nitrate respiration in environments in which the amount of nitrate is extremely low [14]. Previous study [58] also suggests that *nap* may be involved in redox balancing when metabolic rates are high, or when geochemical changes lead to physiological redox imbalances in *Ridgeia* symbionts. Many ε-Proteobacteria species only contain *nap* and are capable of anaerobic nitrate respiration [59]. By comparison, other organisms such as *Shewanella* spp. that inhabit diverse environments have both *nap* and *nar* systems [60]. Respiration via the *nap* system is thought to typically generate less energy than *nar* system [61], although diverse physiological functions have been suggested for bacterial *nap* systems, such as the dissipation of excess reducing equivalents for redox balancing, denitrification, the adaptation to anaerobic growth, and in facilitating nitrate respiration in nitrate-limited environments [62]. Environmental conditions primarily dictate the variation of symbiont nitrogen metabolism in the *Ridgeia* symbioses [58]. Along these lines, nitrate metabolism in siboglinid symbionts appears more complex than previously recognized and might permit adaption to physiological functions or different life stages, although further analysis is warranted to explicitly investigate these hypotheses.

#### Hydrogen oxidation

Similar to symbionts living in *Bathymodiolus* mussels, lucinid bivalve *Loripes* and *Rimicaris* shrimp [35, 63, 64], we were able to identify core enzymes required for hydrogen oxidation, including the group 1 NiFe hydrogenases (*hupL*, *hupS*) within all siboglinid-symbiont genomes (Supplementary Figure S5A). The *hupL* gene predicted to encode the uptake hydrogenase has been previously identified in vent-dwelling *Riftia* endosymbionts [64], as well as in the genome from *Lamellibrachia anaximandri* found in sedimented hydrothermal vent sites [19]. Two additional *Isp-type* hydrogenase genes were identified between the large (*hupL*) and small subunit (*hupS*) hydrogenase genes in the endosymbiont genomes we examined (Supplementary Figure S3A). A phylogenetic analysis indicated that siboglinid *hupL* sequences are closely related to a clade comprising sequences from *Thiothrix* sulfur bacteria (Supplementary Figure S3B) that hypothetically can also use hydrogen as an electron donor [65]. Given that we found evidence for hydrogenotrophy in all endosymbiont genomes we sampled and there have been reports of hydrogen oxidation in other annelid endosymbionts (e.g., the shallow water oligochaete *Olavius algarvensis* sulfur-oxidizing symbionts [66]), the ability to use hydrogen as an additional energy source is probably more widespread than previously suspected.

### Infection process

Although establishing symbiotic associations between tubeworms and symbionts is essential for the holobiont to thrive in deep-sea reducing habitats (Fig. 1d), the molecular mechanisms that underpin the infection process are still largely unknown. For horizontally transmitted symbionts, the infection process can be divided into two steps: colonization of host cells and subsequent migration to the trophosome [12]. Siboglinid symbionts infect the skin of larvae during the post-settlement stage and then travel through host epidermal cells into a mesodermal tissue that later develops into the trophosome. The infection process finishes simultaneously with massive apoptosis of skin tissue in the juvenile stage [8]. Our examination of symbiont genomes reveals several important mechanisms used in the host infection process including genes implicated in adhesion to the host, bacterial secretion systems and virulence, and managing oxidative stress.

Several adhesion-related proteins such as an Ankyrin-like protein and a Fibronectin type III domain were identified in all sampled endosymbiont genomes. The Fibronectin type III domain has only been found to date in vent-dwelling vestimentiferan symbionts [14]. We hypothesize that these gene products may be involved in establishing the initial adhesion of bacteria to host surfaces as siboglinid symbionts are thought to reach their hosts via flagellar- and type IV pili-mediated motility guided by chemotaxis [12]. Type IV pili might also be essential for adhesion and biofilm formation via twitching motility, which also contributes to pathogenesis [67].

Interestingly, a spectacular range of methyl-accepting chemotaxis proteins (MCPs) and their associated chemotaxis protein homologs (*che*) were identified in vent-dwelling and seep-living siboglinid-symbiont genomes (Supplementary note S4). The initial physical encounter between host and symbionts occurs in an extracellular mucous secreted by pyriform glands by newly settled larvae [8]. In mucus matrices, symbionts can attach to the host using extracellular components secreted from symbionts, such as lipopolysaccharide (LPS) and O-antigen, that can mediate direct physical contact between symbionts and their hosts [12, 68].

Whereas many symbionts (e.g., rhizobia, enteropathogenic *Escherichia coli*) use a type III secretion system (T3SS) to avoid phagocytosis and facilitate bacterial invasion into host cells [69], T3SS was lacking in siboglinid symbionts. Instead, the conserved core components of a type II secretion system (T2SS) were identified. Hemolysin and Chitinase exported by the T2SS have been shown to be important for virulence in many pathogens and beneficial microbes (e.g., *Aeromonas veronii* in the leech gut, [70], and *Burkholderia rhizoxinica* in a fungus, [71]). A hemolysin III (*hlyIII*) gene encoding a hemolysin previous reported in *Riftia* symbionts [14, 72] and a gene for a chitinase were identified in all siboglinid symbionts that may enable bacteria to permeabilize the host cells and migrate inter- and intracellularly to newly developed trophosome tissue [12, 14]. Although symbionts are mostly dependent on production of specific toxins, extracellular proteolytic enzymes are also thought to have key roles in host colonization [73] and escape from the host upon host death [13]. We found genes encoding a Chitinase and Collagenase in siboglinid-symbiont genomes, which might assist symbiont migration into host tissues and facilitate toxin diffusion [73]. Furthermore, unlike symbionts of *Bathymodiolus* mussels that contain abundant toxin-related genes [74], only a few *RTX* (repeat motifs in toxins genes) homologs were identified in siboglinid-symbiont genomes. We found little evidence of virulence factors in all siboglinid-symbiont genomes. The only predicted proteins belonging to the virulence category are involved in the production of bacteriocin Colicin V. The Colicin V protein may enable the bacteria to permeabilize the epidermal cells of the host during infection process [14], and it appears to be conserved in all siboglinid symbionts. Genes related to oxidative stress of siboglinid-symbiont genomes may also be of intrest during infection process (Supplementary Note S6).

## Conclusions

To understand how host–symbiont associations adapt to extreme chemosynthetic environments, we compared endosymbiont genomes from tubeworms living in different habitats to identify conserved and variable genomic elements that may, in part, explain the ecological and evolutionary success of a given siboglinid lineage to a particular environment. All siboglinid endosymbionts sampled are predicted to have similar processes related to endosymbiont infection of the host, sulfur metabolism, and hydrogen oxidation. However, vestimentiferan endosymbionts have greater metabolic flexibility of carbon fixation (e.g., Calvin Cycle and rTCA) in energy-rich reducing habitats, such as vents and seeps. Comparatively, the more diminutive and less ecologically dominant frenulates lack rTCA mechanisms as well as some of the nitrate reductase mechanisms (e.g., *narGHIJ* and *napABC*) found in other siboglinids. Importantly, our findings suggest that variation in carbon and nitrogen metabolism, rather than sulfur metabolism, may drive host–symbiont interactions that allow holobionts to colonize and adapt to different chemosynthetic environments. These results likely extend to other taxa (e.g., deep-sea mussels, clams, snails and shrimp, or shallow water bivalves) that harbor chemosynthetic endosymbionts.

## Electronic supplementary material

Supplementary materials
