# Supplementary material for: Endosymbiont genomes yield clues of tubeworm success
Source: ISME J. 2018 Jul 18;12(11):2785–95. doi: 10.1038/s41396-018-0220-z (PMC6194059; doi:10.1038/s41396-018-0220-z)
Supplement: Supplementary file 1 — Supplementary materials [file 41396_2018_220_MOESM1_ESM.docx]

**Supplementary Materials for:**

**Genomic clues of tubeworm success**

Yuanning Li ^*^, Mark R. Liles, Kenneth M. Halanych ^*^

### Supplementary note S1: General genomic features

The sequencing depths (~ 20X) of the novel genomes reported here were similar or even better than other assemblies (Table 1). *Escarpia* (23 contigs) and *Galathealinum* (14 contigs) are more complete than either *Seepiophila* (323 contigs) or *Lamellibrachia* (337 contigs) endosymbiont assemblies. Notably, the GC-content of seep-dwelling vestimentiferan symbionts (~ 54%) was slightly lower than vent-dwelling ones (~ 58%). The GC-content of heterotrophic *Osedax* bacteria was ~ 43%, whereas the *Galathealinum* symboint genome was even lower with a content of 38.9%. The GC content of bacterial genomes has been thought to associate with genome size, oxygen and nitrogen exposure and habitats (Bohlin *et al*., 2010). The base composition in GC-rich bacterial genomes might reflect stronger selective forces than AT-rich genomes (Bohlin *et al*., 2010). This correspondence in siboglinid symbionts might reflect that host-symbiont specificity in major siboglinid lineages (Schulze and Halanych 2003), an adaptation of symbiont genomes to more-reducing environments, or possibly both. Consistent with other siboglinid assemblies, we were unable to close and circularize these symbiont genomes even with such high sequencing coverage due to the presence of repetitive regions (Perez and Juniper, 2016). Either situation would offer an explanation on reported difficulties in assembling siboglinid symbiont genomes in general.

**Supplementary note S2: Phylogenetics**

The dataset for phylogenetic analysis using 16S rDNA consisted of 1,458 nucleotide positions. All four endosymbionts belonged to sulfur-oxidizing bacteria within γ-Proteobacteria (Supplementary Figure S1). Although four siboglinid endosymbionts were collected from around the same seep locality in GOM, they harbored different ribotypes of endosymbionts. *Escarpia, Seepiophila* and *Lamellibrachia* symbiont sequences were clustered as a well-supported clade with other seep-living vestimentiferans (bs = 96%) with ~95% sequence similarity to vent-dwelling vestimentiferans, whereas *Galathealinum* was most close related with other frenulate species with a strong nodal support (bs = 100%) with ~ 90% sequence similarity with other siboglinid symbionts. Within seep-dwelling vestimentiferans endosymbionts, three monophyletic groups were identified in previous studies (McMullin *et al*., 2003). *Lamellibrachia* and *Seepiophila* shared the identical 16S rRNA gene sequences and clustered within “group 2” endosymbionts. However, *Escarpia* endosymbiont was different than *Lamellibrachia* and *Seepiophila* symbionts (differing by 26 bp) and formed its own clade instead of affiliating with previous sequenced *Escarpia* symbionts (Supplementary Figure S1).

Coevolution between host and their symbiont was not expected to arise in siboglinids because symbionts are acquired through horizontal transmission mode. Here, we used phylogenomic analyses to assess the topological patterns between host and symbiont trees. Two datasets were generated here: the host dataset contained 289 orthologous groups (OGs) which derived from the study of (Li *et al*., 2017); the symbiont dataset comprised of 552 highly conserved OGs after several steps of filtering.

**Supplementary note S3: Sulfur metabolism**

Sulfur-oxidizing endosymbiont bacteria oxidize reduced inorganic sulfur compounds to provide energy needed for hosts (Cavanaugh *et al*., 2006). Similar to *Riftia* endosymbionts (Markert *et al*., 2011), all sequenced tubeworm symbiont genomes possess the APS reductase pathway including APS reductase (*AprA*/*AprB*), ATP sulfurylase (*dsrA*/*dsrB*) and ATP sulfurylase (*sopT*). Furthermore, components of thiosulfate-oxidizing Sox enzyme system (*soxABXYZ*) were also identified, whereas *soxCD* homolog, was missing in all siboglinid symbionts. Moreover, since sulfide, instead of thiosulfate, is inferred to be the preferred energy source for vent-living vestimentiferan symbionts (Markert *et al*., 2011; Gardebrecht *et al*., 2012), the presence of Sox system in symbiont genomes suggests that thiosulfate oxidation might serve as an alternative sulfur metabolic pathway while sulfide is at minimum levels. The *soxCD* homolog is absent in most sulfur globule-forming organisms, such as *Allochromatium vinosum* (Weissgerber *et al*., 2011). Among these organisms, the Sox complex is involved in the oxidation of thiosulfate to sulfate and then store sulfur globules as intermediates (Welte *et al*., 2009). Since sulfur globules have been isolated in *Riftia* symbionts (Markert *et al*., 2011), formation of intracellular sulfur globules for sulfur storage might be common across siboglinids.

**Supplementary note S4: Chemotaxis and motility**

Motility and chemotaxis are essential for many pathogenic or symbiotic species during the free-living stage to allow colonization and infection of a host (Wadhams and Armitage, 2004). For example, chemotaxis can guide *Vibrio anguillarium* to the surface tissue of fish and *Vibrio fischeri* to the squid light organs (Nyholm and Mcfall-Ngai, 2004). Although previous Cand. *Endoriftia persephone* metagenomic analysis showed considerable chemoreception and motility machinery (Robidart *et al*., 2008), detailed molecular pathway has not been well characterized. A spectacular range of methy-accepting chemotaxis proteins (MCPs) and their associated chemotaxis protein homologs (*che*) were identified in vent-living (Gardebrecht *et al*., 2012; Perez and Juniper 2016) and seep-dwelling siboglinid symbiont genomes. Most environmental signals are sensed by receptors (MCPs) and MCPs subsequently regulate the activity of the histidine protein kinase *cheA* through the linker protein *cheW* (Zusman *et al*., 2007). Although chemosensory pathways can usually be identified by a specific *cheA* (Zusman *et al*., 2007), several conserved chemosensory co-located gene clusters were identified in most siboglinid symbiont genomes (Supplementary Figure S7), although these gene clusters were not found in *Lamellibrachia* and *Seepiophila* symbiont genomes probably due to highly fragmented genome assembly. Although the actual function is still unknown, the gene arrangement of cheI cluster in tubeworm symbionts is highly similar to cheIII system in *M. xanthus* and cheII system in *Rhizobium leguminosarum* (Miller *et al*., 2007) which has a minor effect on bacterial motility. The cheII system identified in siboglinid symbiont genomes is highly similar to bivalve symbiont *Solemya velum*, both contain *pilGHIJ-cheAW* gene clusters, which are known to control twitching motility in other bacteria (Dmytrenko *et al*., 2014). For cheIII chemosensory system, chemotaxis protein homologs are co-located with flagellar biosynthesis gene clusters and flagellar motility proteins (*motA* and *motB*). As shown in *E. coli*, *motA* and *motB* are required for generation of rotation force during flagellar movement (Stolz and Berg, 1991). Thus, the cheIII system identified here might have a function in controlling flagellar motility guided by chemotaxis for siboglinid symboints. Noticeable, all sequenced symbiont genomes contained the full complement of genes that were indispensable for a functional flagellum and type IV pili (T4P), plus a number of accessories or duplicated components. Although a flagellum was not detected in *Riftia* symbionts while in the trophosome, it might be present in the free-living life stage and genes associated with flagellar synthesis are no longer expressed after infection (Millikan *et al*., 1999; Robidart *et al*., 2008). Altogether, motility of siboglinid bacteria was probably mediated by the flagellar motility and extension, retraction of type IV pili T4P guided by chemotaxis.

**Supplementary note S5: Oxidative stress**

Consistent with a previous description of the oxidative stress response in *Riftia* (Gardebrecht *et al*., 2012), Superoxide dismutase (*FeSOD*) and Alkylhydroperoxide reductase (*ahpC*) genes were identified in all vestimentiferan symbiont genomes in this study. These gene products likely help symbionts overcome host cellular immune responses (Bright and Bulgheresi, 2010). However, *FeSOD* was lacking in the *Galathealinum* symbiont genome. Furthermore, a Rubrerythrin (*rbr)* gene was detected in all siboglinid symbiont genomes, and *rbr* has been suggested to play a role in protection against hydrogen peroxide-mediated oxidative stress and damage (Konig *et al*., 2016). In addition, multiple gene copies of antioxidant proteins, such as Cytochrome c peroxidase and Thiol peroxidase, were also identified in siboglinid sulfur-oxidizing symbionts.

**References**

Bohlin J, Snipen L, Hardy SP, Kristoffersen AB, Lagesen K, Donsvik T *et al.* (2010). Analysis of intra-genomic GC content homogeneity within prokaryotes. *Bmc Genomics* **11**.

Cavanaugh CM, McKiness ZP, Newton IL, Stewart FJ. (2006). Marine chemosynthetic symbioses. *The prokaryotes*. Springer. pp 475-507.

Dmytrenko O, Russell SL, Loo WT, Fontanez KM, Liao L, Roeselers G *et al.* (2014). The genome of the intracellular bacterium of the coastal bivalve, *Solemya velum*: a blueprint for thriving in and out of symbiosis. *BMC Genomics* **15:** 924.

Gardebrecht A, Markert S, Sievert SM, Felbeck H, Thurmer A, Albrecht D *et al.* (2012). Physiological homogeneity among the endosymbionts of *Riftia pachyptila* and *Tevnia jerichonana* revealed by proteogenomics. *ISME J* **6:** 766-776.

Konig S, Gros O, Heiden SE, Hinzke T, Thurmer A, Poehlein A *et al.,* (2016). Nitrogen fixation in a chemoautotrophic lucinid symbiosis. *Nat Microbiol* **2:** 16193.

Li Y, Kocot KM, Whelan NV, Santos SR, Waits DS, Thornhill DJ *et al.,* (2017). Phylogenomics of tubeworms (Siboglinidae, Annelida) and comparative performance of different reconstruction methods. *Zool Scripta* **46:** 200-213.

Markert S, Gardebrecht A, Felbeck H, Sievert SM, Klose J, Becher D *et al.* (2011). Status quo in physiological proteomics of the uncultured Riftia pachyptila endosymbiont. *Proteomics* **11:** 3106-3117.

McMullin ER, Hourdez S, Schaeffer SW, Fisher CR. (2003). Phylogeny and biogeography of deep sea vestimentiferan tubeworms and their bacterial symbionts. *Symbiosis* **34:** 1-41.

Miller LD, Yost CK, Hynes MF, Alexandre G. (2007). The major chemotaxis gene cluster of Rhizobium leguminosarum bv. viciae is essential for competitive nodulation. *Mol Microbiol* **63:** 348-362.

Millikan DS, Felbeck H, Stein JL. (1999). Identification and characterization of a flagellin gene from the endosymbiont of the hydrothermal vent tubeworm *Riftia pachyptila*. *Appl Environ Microbiol* **65:** 3129-3133.

Nyholm SV, Mcfall-Ngai MJ. (2004). The winnowing: Establishing the squid-*Vibrio* symbiosis. *Nat Rev Micro* **2:** 632-642.

Parks DH, Imelfort M, Skennerton CT, Hugenholtz P, Tyson GW. (2015). CheckM: assessing the quality of microbial genomes recovered from isolates, single cells, and metagenomes. *Genome Res* **25:** 1043-1055.

Perez M, Juniper SK. (2016). Insights into Symbiont Population Structure among Three Vestimentiferan Tubeworm Host Species at Eastern Pacific Spreading Centers. *Appl Environ Microbiol* **82:** 5197-5205

Robidart JC, Bench SR, Feldman RA, Novoradovsky A, Podell SB, Gaasterland T *et al.,* (2008). Metabolic versatility of the *Riftia pachyptila* endosymbiont revealed through metagenomics. *Environ Microbiol* **10:** 727-737.

Schulze A, Halanych KM. (2003). Siboglinid evolution shaped by habitat preference and sulfide tolerance. *Hydrobiologia* **496:** 199-205.

Stolz B, Berg HC. (1991). Evidence for Interactions between Mota and Motb, Torque-Generating Elements of the Flagellar Motor of *Escherichia coli*. *J Bacteriol* **173:** 7033-7037.

Wadhams GH, Armitage JP. (2004). Making sense of it all: bacterial chemotaxis. *Nat Rev Mol Cell Biol* **5:** 1024-1037.

Weissgerber T, Zigann R, Bruce D, Chang YJ, Detter JC, Han C *et al.,* (2011). Complete genome sequence of *Allochromatium vinosum* DSM 180(T). *Stand Genomic Sci* **5:** 311-330.

Welte C, Hafner S, Kratzer C, Quentmeier A, Friedrich CG, Dahl C. (2009). Interaction between Sox proteins of two physiologically distinct bacteria and a new protein involved in thiosulfate oxidation. *FEBS Lett* **583:** 1281-1286.

Zusman DR, Scott AE, Yang Z, Kirby JR (2007). Chemosensory pathways, motility and development in Myxococcus xanthus. *Nat Rev Microbiol* **5:** 862-872.

Supplementary Figure S1: Maximum likelihood inference phylogeny of known γ-Proteobacteria siboglinid symbionts based on 1458-bp 16S rRNA gene sequences. Bootstrap values indicated next the relevant node. Red indicates the taxa sequenced in this study.

Supplementary Figure S2: Presence/absence of 106 single-copy universal bacterial genes used to estimate genome completeness. Red indicates a marker gene was not detected, green indicates a single copy of the marker was recovered and yellow indicates multiple copies were recovered.

Supplementary Figure S3: (A) Gene clusters and (B) phylogenetic analysis of group 1 membrane-bound Ni, Fe hydrogenase large subunit inferred from maximum likelihood using RAxML. Bootstrap values indicated next the relevant node. Sequences in red are taxa derived from this study.

Supplementary Figure S4: Functional protein composition of seven siboglinid symbiont genomes, annotated genes were assigned to RAST subsystem category and then plotted for comparison.

Supplementary Figure S5: Cophylogeny analysis of (A) bacterial symbionts and their (B) associated siboglinid hosts and inferred with maximum likelihood. All nodes are 100 bootstrap values.

Supplementary Figure S6: Multiple chemosensory gene clusters in siboglinid symbionts. Chemotaxis pathways in were identified by computer searches (BLAST) for genes that encode CheA homologues and other chemotaxis proteins.

Supplementary Table S1.

Completeness check for each symbiont genome assembly based on 577 lineage-specific (544 for *Galathealinum*) single copy marker sets (Parks *et al*., 2015) from 112 γ-proteobacteria genomes generated from Checkm.

| Bin ID | Marker sets | Present | Duplicates | Missing | Completeness% | Contamination% |
| --- | --- | --- | --- | --- | --- | --- |
| *Riftia* | 577 | 491 | 7 | 80 | 87.81 | 1.74 |
| *Ridgeia* | 577 | 564 | 4 | 10 | 98.41 | 0.7 |
| *Tevnia* | 577 | 540 | 5 | 32 | 93.85 | 1.05 |
| *Seepiophila* | 577 | 562 | 5 | 15 | 97.64 | 1.74 |
| *Lamellibrachia* | 577 | 551 | 6 | 21 | 96.19 | 2.09 |
| *Escarpia* | 577 | 566 | 4 | 7 | 99.18 | 0.7 |
| *Galathealinum* | 544 | 535 | 5 | 4 | 99.44 | 1.23 |

Supplementary Table S2. Average amino acids identity (AAI) and Average nucleotide identity (ANI) values calculated between seven siboglinid symbiont genomes.

| AAI/AAI | *Riftia* | *Ridgeia* | *Tevnia* | *Lamellibrachia* | *Seepiophila* | *Escarpia* | *Galathealinum* |
| --- | --- | --- | --- | --- | --- | --- | --- |
| *Riftia* | - |  |  |  |  |  |  |
| *Ridgeia* | 97.51/97.37 | - |  |  |  |  |  |
| *Tevnia* | 99.50/99.8 | 97.89/97.30 | - |  |  |  |  |
| *Lamllibrachia* | 68.90/78.99 | 69.75/78.68 | 69.61/79.00 | - |  |  |  |
| *Seepiophila* | 68.94/79.01 | 69.67/78.71 | 69.68/78.96 | 99.94/100 | - |  |  |
| *Escarpia* | 68.60/78.77 | 69.60/78.67 | 69.35/78.82 | 95.36/95.36 | 95.33/94.88 | - |  |
| *Galathealinum*^a^ | 50.60/- | 51.43/- | 51.02./- | 51.36/- | 51.30/- | 50.99 | - |

^a^ ANI values of *Galathealinum* are below detection from the algorithm and thus the results are not shown.
